# A rare case of a large low-grade appendicular mucinous neoplasm causing compressive symptoms

**DOI:** 10.1093/jscr/rjac111

**Published:** 2022-04-13

**Authors:** John R Ekblad, Sidra B Bhuller, John Weaver, Michael E Bertocchi

**Affiliations:** Department of Surgery, Sky Ridge Medical Center, Lone Tree, CO, USA; Department of Surgery, Sky Ridge Medical Center, Lone Tree, CO, USA; Department of Surgery, Sky Ridge Medical Center, Lone Tree, CO, USA; Department of Surgery, Sky Ridge Medical Center, Lone Tree, CO, USA

**Keywords:** abdominal mass, appendix, LAMN, low-grade, mucinous neoplasm, mucocele

## Abstract

Primary neoplasm of the appendix is often diagnosed incidentally after an appendectomy. Low-grade appendiceal mucinous neoplasms (LAMNs) make up a small portion of these neoplasms. We present a rare case of a patient with a slow-growing LAMN causing urinary retention and constipation. The mass was initially found incidentally 25 years prior, but the patient declined further workup since he was asymptomatic at that time. The patient experienced progressively worsening abdominal discomfort related to urinary retention and difficulty in evacuating his bowels. Imaging identified a large abdominal mass (19.3 × 8.7 × 13.5 cm). The mass was surgically resected. Pathology was consistent with a LAMN. In general, an incidental finding of an abdominal mass should be further investigated regardless of symptomology. Patients should be educated about the potential of malignancy and the need for a major abdominal surgery in the future if they choose not to have a mass further evaluated.

## INTRODUCTION

Primary neoplasm of the appendix is found in 2% of patients undergoing surgical appendectomy; of those, appendiceal mucinous neoplasms (AMN) are found in 0.2–1.4% [1, 2]. AMNs range in presentation from simple mucoceles to complex pseudomyxoma peritonei (PMP) [2]. World Health Organization 2019 classification divides neoplastic appendiceal lesions into serrated polyps, hyperplastic polyps, low-grade appendiceal mucinous neoplasms (LAMNs), high-grade AMNs and mucinous adenocarcinomas [1]. Stage and histology features of the tumor determine treatment [2]. Treatment is typically surgical, but the extent of resection is not universally agreed upon [1–3]. Given the rarity of AMNs and controversies surrounding their treatment, it is important for surgeons to be aware of how AMNs can present, their workup, treatment modalities and their long-term follow-up.

Here, we present a patient with a rare case of a LAMN, which presented as a slow-growing abdominal mass with compressive urinary and bowel symptoms.

## CASE REPORT

A healthy, active 47-year-old male with history of tobacco use presented with a slow-growing abdominal mass. This mass was initially found incidentally on imaging 25 years ago; however, the patient chose not to undergo further investigation or intervention at that time since he was asymptomatic. Presently, patient returned with progressively worsening urinary retention and constipation associated with progressive abdominal distension and intermittent abdominal discomfort. The patient denied hematuria, urinary incontinence, loss of appetite, weight loss, nausea, vomiting, diarrhea and dyspepsia. The patient never had a colonoscopy. Physical exam was remarkable for a large, firm and immobile mass that was located throughout the entire lower abdomen.

**Figure 1:**
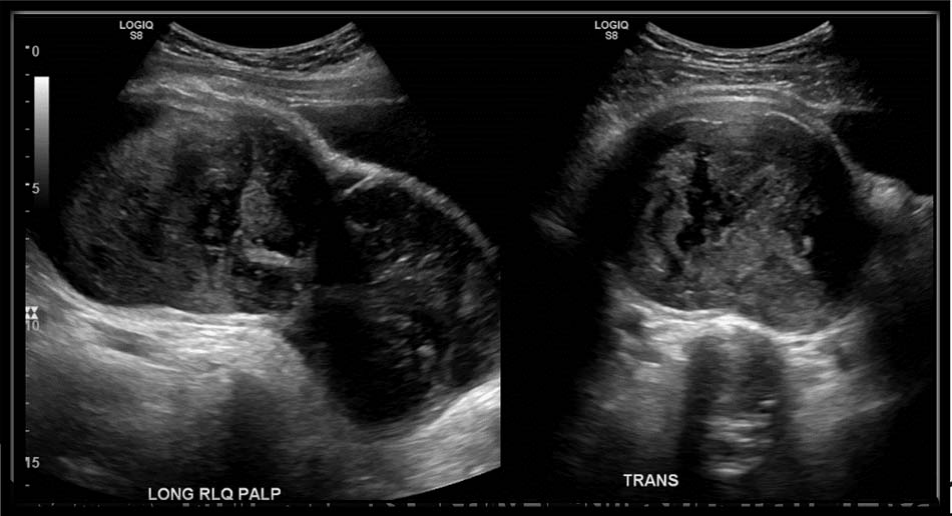
US of right lower quadrant visualizing the hypo-vascular abdominal mass longitudinally and transversely; mass measured 18.5 × 8.1 × 11 cm in the right hypogastric region with only a small amount of visible internal flow on color Doppler.

**Figure 2:**
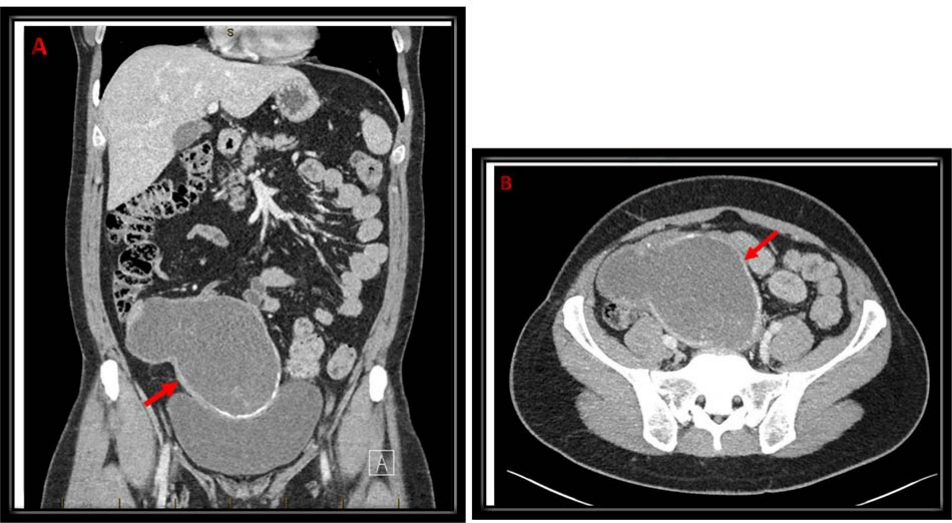
Coronal (**A**) and transverse (**B**) cuts of CT abdomen/pelvis demonstrating a large complex mass (red arrow) extending from the central pelvis to the right lower quadrant of the abdomen with some calcifications at the inferior and medial edges with some internal calcifications.

An abdominal ultrasound (US) was performed and showed a hypo-vascular mass in the right hypogastric region (Fig. 1). Abdomen and pelvis computed tomography (CT) scan with intravenous contrast identified a large complex mass (19.3 × 8.7 × 13.5 cm) with a partially calcified soft tissue rim with some internal calcifications. The mass extended from the central pelvis to the right lower quadrant of the abdomen (Fig. 2). The mass appeared to be attached to the cecum and no normal appendix was identifiable on imaging. There was no free fluid or evidence of metastasis. The tumor was compressing the bladder and sigmoid colon on CT scan, which explains the patient’s urinary and bowel symptoms.

Given the patient’s situation, the patient was advised to have the tumor surgically resected. A barium enema and cystourethrogram was completed before the operation to better visualize the extent to which surrounding structures were involved; no communication or fistulas were found. An elective exploratory laparotomy with complete resection of the intraabdominal mass was performed. The mass along with a portion of the cecum were sent to pathology for analysis. Intraoperatively, the mass was noted to contain gelatinous material. Some mucin seeding was noted on the recto-sigmoid junction serosa and bladder wall but no resections were performed. The patient tolerated the procedure well and his recovery was uncomplicated.

**Figure 3:**
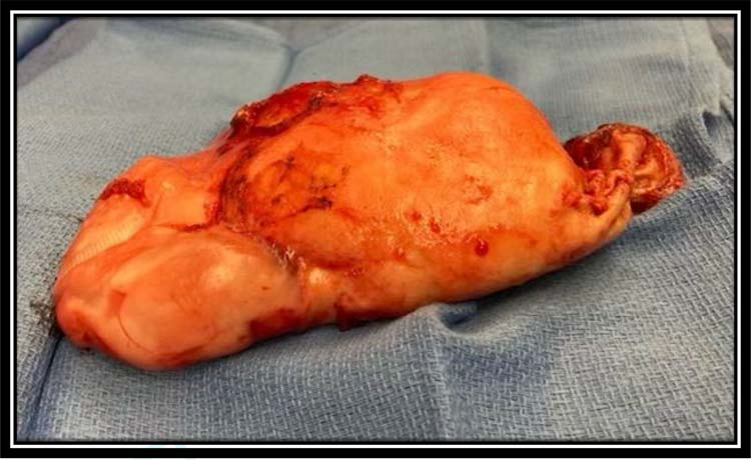
Resected LAMN gross specimen; brown to tan in color, weighed 264 g and measured 17.5 × 11.0 × 4.0 cm.

On pathology, the mass was noted to be a mucinous neoplasm consistent with LAMN. The tumor appeared brown to tan, weighed 264 g and measured 17.5 × 11.0 × 4.0 cm (Fig. 3). The LAMN was noted to be invading the cecal serosa and muscularis propria with extension to within 1 mm of the cecal resection margin. Primary tumor was staged as pT4a and no lymph nodes were collected or identified. No features of invasive adenocarcinoma were identified. The patient was offered HIPEC, given positive margins and mucin seeding noted intraoperatively; however, the patient elected not to undergo any further adjuvant therapies like HIPEC, given the slow growth of tumor over 25 years without any high-grade features. The patient has been doing well since the procedure and has not had reoccurrence of the tumor or pseudotumor myxoma at 6 months since resection of the LAMN.

## DISCUSSION

LAMN most often presents like an acute appendicitis due to distension of the appendix, and the diagnosis is made post-operatively during histologic examination of the appendix [1, 4, 5]. Less commonly, symptoms may include abdominal pain, a palpable mass, urinary retention, constipation or difficulty in evacuating stool, gastrointestinal bleeding and/or abdominal distention secondary to PMP [6]. Appendiceal neoplasms can also be found incidentally on imaging [6].

CT scan is the imaging of choice to further evaluate the tumor since it can help delineate mass effect and metastatic disease if present [7, 8]. LAMNs appear to be cystic, fluid filled and thin-walled, with or without the presence of calcifications within the walls [7]. Other useful imaging techniques include magnetic resonance imaging, US and colonoscopy [7]. Lab workup should also include tumor markers such as carcinoembryonic antigen and carbohydrate antigen 19-9 [9, 10].

Treatment for an AMN depends on the size and surrounding structures’ involvement. A small LAMN confined to the appendix can be definitively treated with open or laparoscopic appendectomy; larger LAMN will require an open appendectomy or laparotomy [7, 8, 11]. If the tumor involves the proximal portion of the appendix making it difficult to remove the appendix alone, an ileocecal resection or removal of part of the cecum may be indicated [7, 12]. Care must be taken when dissecting and extracting the appendicular tumor to prevent perforation. Leakage of mucinous contents from a perforation could lead to seeding of the LAMN into the peritoneum, putting the patient at risk for PMP [7]. A laparoscopic approach has been shown to have a higher risk of rupture compared to an open approach when extracting these tumors [7, 11]. Histologic examination confirms the diagnosis of a LAMN. Histology will show low-grade cytologic atypia along with loss of the muscularis mucosa or fibrosis of the submucosa [11]. Each patient should have an individualized review with a multidisciplinary committee to determine if adjuvant chemotherapy or HIPEC will be beneficial [13].

## CONCLUSION

In general, an incidental finding of an abdominal mass should be further investigated regardless of symptomology. Patients should be educated about the potential of malignancy and need for a major abdominal surgery in the future if they chose not to have their mass worked up. AMNs are rare and can have a variable presentation; they should be considered in the differential diagnosis for any appendiceal mass.

## CONFLICT OF INTEREST STATEMENT

None declared.

## FUNDING

None.

## DISCLAIMER

This research was supported (in whole or in part) by HCA Healthcare and/or an HCA Healthcare affiliated entity. The views expressed in this publication represent those of the authors and do not necessarily represent the official views of HCA Healthcare or any of its affiliated entities.
